# Developmental history, energetic state and choice impulsivity in European starlings, *Sturnus vulgaris*

**DOI:** 10.1007/s10071-019-01254-5

**Published:** 2019-03-06

**Authors:** Jonathon Dunn, Clare Andrews, Daniel Nettle, Melissa Bateson

**Affiliations:** Centre for Behaviour and Evolution and Institute of Neuroscience, Newcastle University, Newcastle, UK

**Keywords:** Impulsivity, Energetic state, Telomeres, Starlings, *Sturnus vulgaris*, Avian cognition

## Abstract

Impulsivity—the extent to which a reward is devalued by the amount of time until it is realized—can be affected by an individual’s current energetic state and long-term developmental history. In European starlings (*Sturnus vulgaris*), a previous study found that birds that were lighter for their skeletal size, and birds that had undergone greater shortening of erythrocyte telomeres over the course of development, were more impulsive as adults. Here, we studied the impulsivity of a separate cohort of 29 starlings hand-reared under different combinations of food amount and begging effort. The task involved repeated choice between a key yielding one pellet after 3 s and another key yielding two pellets after 8 s. Impulsivity was operationalised as the proportion of choices for the short-delay option. We found striking variation in impulsivity. We did not replicate the results of the previous study concerning developmental telomere attrition, though combining all the evidence to date in a meta-analysis did support that robustness of that association. We also found that early-life conditions and mass for skeletal size interacted in predicting impulsivity. Specifically, birds that had experienced the combination of high begging effort and low food amount were less impulsive than other groups, and the usual negative relationship between impulsivity and body mass was abolished in birds that had experienced high begging effort. We discuss methodological differences between our study and studies that measure impulsivity using an adjusting-delay procedure.

## Introduction

Every day animals make many choices between pursuing a larger future reward and pursuing a more immediate smaller one. The extent to which rewards are devalued as the time until their realization increases will henceforth be referred to as impulsivity (although note this term is also used elsewhere with other meanings, and what we refer to as impulsivity is also referred to elsewhere as time preference or delay discount rate). Impulsivity in decision-making is context and state dependent, that is, it is influenced not only by the objective value of the rewards involved, but by the state of the individual, and their ecological milieu (Daly and Wilson 2005; Fawcett et al. 2012; Lempert and Phelps 2016). Two kinds of state influences on impulsivity have been identified. First, there are short-term energetic influences: animals, including humans, often become more impulsive as their energetic reserves decrease or the time since they last ate increases (Snyderman 1983; Wang and Dvorak 2010; Bateson et al. 2015; Mayack and Naug 2015; Allen and Nettle 2019). Second, there may be influences of long-term developmental history. In humans, coming from a more deprived childhood background is robustly associated with increased impulsivity (Paál et al. 2015; Allen and Nettle 2019). In non-human systems, the evidence is more equivocal: manipulating developmental conditions has been found in some studies to affect impulsivity in adulthood (early stress leading to greater impulsivity; Gondré-Lewis et al. 2016), but in other studies to affect other behavioural traits but not impulsivity (Lovic et al. 2011; Brydges et al. 2015).

In a recent study of adult European starlings (*Sturnus vulgaris*), Bateson et al. (2015) showed that individuals’ levels of impulsivity were additively predicted by their current energetic state (greater mass for skeletal size being associated with less impulsivity), and the developmental telomere attrition that they had experienced (greater developmental telomere attrition being associated with greater impulsivity). Developmental telomere attrition refers to the extent to which the telomeres (DNA–protein caps on chromosomes) shorten over the developmental period. Greater developmental telomere attrition is associated with more adverse rearing conditions (Nettle et al. 2013, 2015b; Boonekamp et al. 2014; Reichert et al. 2014), and shorter subsequent adult lifespan (Heidinger et al. 2012; Boonekamp et al. 2014; Wilbourn et al. 2018). Thus, the greater impulsivity observed by Bateson et al. (2015) in individuals that had undergone greater developmental telomere attrition may have reflected the poorer state and lower future life expectancy of those individuals. The birds studied by Bateson et al. (2015) had been subject to a developmental manipulation (cross-fostering into either small or large broods). The developmental treatments affected developmental telomere attrition, which in turn predicted adult impulsivity, but there was no significant direct association between developmental treatment and impulsivity. This may be because developmental telomere attrition integrates across many different components of developmental experience, and captures the variation in their effects on the individuals’ states, and thus provides a better individual-level summation of long-term organismal state.

A subsequent replication study (Nettle et al. 2015a) found only weak evidence for the pattern seen in the initial study; although the effects of mass and developmental telomere attrition were in the same directions, tests against the null hypothesis of zero association were not significant by conventional criteria. Both earlier studies measured impulsivity using an adjusting-delay procedure (Mazur and Biondi 2009). Birds are trained that pecking one key produces a small reward after a fixed short delay, and another a larger reward after a different delay, the ‘adjusting’ delay. Over a series of trials, the length of the adjusting delay is varied in response to the birds’ choices to estimate the point at which the bird is indifferent between the two options, and hence its degree of impulsivity. Variants of the adjusting-delay procedure are widely used (Craig et al. 2014). However, a potential issue with it is that the adjusting delay oscillates over the course of the experiment, and usually within each experimental session. Hence, the adjusting delay is, in each subject’s experience, not only longer than the fixed delay, but also variable, whereas the other delay is fixed. Animals are typically not indifferent between two options with the same average value but different variances (Kacelnik and Bateson 1996; 2002). Thus, the indifference point in the adjusting-delay procedure could be affected not just by the individual’s impulsivity, but also by the extent to which they are averse to (or prefer) a variable as compared to a fixed option (known as their risk preference). Of two individuals with the same degree of impulsivity but different risk preferences, the one more averse to risk might choose the fixed option more often, causing the adjusting delay to shorten, and hence the individual’s apparent impulsivity to be greater.

Bateson and Kacelnik (1996) defended adjusting-delay procedures on the grounds that the rate of change of the adjusting delay is slow and the increments small; that the parameter values obtained suggest that the birds perceive the two options as fixed; and that when the adjusting delay is fixed at the calculated indifference point, birds continue to be indifferent between the two options. Nonetheless, the issue remains pertinent in view of a recent study of starlings that found risk preference to be associated with developmental telomere attrition: birds with greater developmental telomere attrition were also more averse to variability (Andrews et al. 2018). Thus, there is at least a possibility that the impulsivity measures in the earlier studies may have been somewhat conflated with individual differences in risk preference, which might in turn have been related to developmental telomere attrition (although the developmental telomere attrition–risk aversion association was not measured in those particular individuals).

Here, we carried out a further study of impulsivity in a separate cohort of starlings. Instead of the adjusting-delay procedure, we used an impulsivity paradigm based on the strength of partial preference. In this paradigm, both options are fixed and hence the issue of variability or risk does not arise. We gave repeated simultaneous choices between the same small short-delay and larger long-delay options, and assumed that the more strongly an individual prefers one over the other, the greater will be the proportion of that option chosen over the course of many trials. Although this procedure is not typically used for impulsivity in animals, the equivalent is used in humans, where it is known as the ‘fixed-alternative’ design (Rung et al. 2018). Moreover, the exactly analogous design is common in the study of risk preference in animals; most studies operationalise risk preference in terms of the strength of partial preference between two unchanging options, rather than using an adjusting procedure to estimate an indifference point (Kacelnik and Bateson 1996; Andrews et al. 2018).

The cohort of starlings used in the present study were hand-reared using a two-by-two factorial developmental design, in which birds received either ad lib or restricted early-life food supply, and were required to make either a low or high level of begging effort. Both amount of food and level of begging effort separately and additively affected developmental telomere attrition (Nettle et al. 2017). This was the cohort in which risk preference has already been studied, and is related to developmental telomere attrition (Andrews et al. 2018). Our objectives were as follows. First, we examined whether the findings of Bateson et al. (2015) that developmental telomere attrition and body condition (i.e. mass for skeletal size) additively predicted impulsivity also hold in this cohort with the fixed-alternative measure described above. Second, we formally synthesized the findings from these birds and those of the two earlier studies (Bateson et al. 2015; Nettle et al. 2015a) in a meta-analysis. Third, we investigated the direct effects of developmental treatments (the amount of food and the level of begging effort) on impulsivity in the current cohort of birds. Although the two previous starling studies (Bateson et al. 2015; Nettle et al. 2015a), in line with evidence from other systems (Lovic et al. 2011; Brydges et al. 2015), found no direct effects of developmental treatments on impulsivity, in the present cohort of starlings, there is other evidence for direct treatment effects on adult behavioural phenotype (Neville et al. 2017; Gott et al. 2018). This may be because the developmental treatments in the current birds, who were hand-reared, were better controlled than earlier cross-fostering manipulations. In particular, we have found that birds from this cohort that had to make higher begging effort during development maintained lower body condition in adulthood, and also showed a different pattern of foraging effort for a given level of body condition (Dunn et al. 2018). Thus, any associations between body condition and impulsivity might be moderated by early-life begging effort.

## Methods

### Subjects

Our subjects were from a cohort of 32 European starlings (16 males), hand-reared under a developmental manipulation as described in detail elsewhere (Nettle et al. 2017). Briefly, quartets of nestlings were taken from eight nest boxes 5 days after hatching. One member of each quartet was assigned at random to each of four treatment groups, representing the possible combinations of food Amount (Plenty or Lean) and begging Effort (Easy or Hard). The two ‘Plenty’ groups (Plenty-Easy and Plenty-Hard) received nine feeds to satiation each day. For each feeding visit, birds in the Lean groups (Lean-Easy and Lean-Hard) were restricted to a percentage of the mean amount consumed by the corresponding Plenty group on the most recent feed. Initially, this was 70% but was adjusted over the manipulation to track the poorest growth curves observed in wild nests, averaging 73% overall. The Easy groups (Plenty-Easy and Lean-Easy) were visited only for the nine feeds each day. The Hard groups (Plenty-Hard and Lean-Hard) were visited for an additional nine sham feeds, where they were stimulated to beg but no food was delivered. Each sham feed lasted two minutes, a similar duration to a real feed. Thus, birds in the Hard treatment begged around twice as much as those in the Easy treatment each day. The developmental manipulation continued until day 15 post-hatching, after which all birds were fed ad libitum. Once they fledged and were feeding themselves, they were kept in two indoor mixed-treatment aviaries (215 × 340 × 220 cm; ~ 18C, 40% humidity; 15L:9D light cycle). Tarsus length was measured on day 56 of life with digital callipers; values used are the mean of two replicate measurements from each leg. Birds were genetically sexed after the manipulation, and unfortunately the sex ratios were not well balanced across groups (Plenty-Easy: 4:4; Plenty-Hard: 7:1; Lean-Easy: 0:8; Lean-Hard: 5:3). To control for the confounding of sex with treatment group, we included sex as an additional predictor in the statistical models.

### Developmental telomere attrition

Relative erythrocyte telomere length was assessed from DNA extracted from day-5, -15 and -56 blood samples, using the qPCR method, which provides an estimate of relative mean telomere length in the form of a T/S ratio (the ratio of the abundance of the telomeric sequence in the DNA sample to the abundance of a control gene). DTA was characterised by calculating the variable ΔTL. This was based on the difference between the T/S ratios on day 56 and day 5, and was standardized using the method of Verhulst et al. (2013), which corrects for regression to the mean. A negative value of ΔTL indicates a greater degree of developmental telomere attrition than the average bird in the sample, and a more positive value indicates a lesser degree of developmental telomere attrition, but not telomere lengthening (all birds showed telomere shortening over development). Owing to failed assays, we did not have telomere information for five birds. As previously reported (Nettle et al. 2017) both Amount and Effort had significant additive effects on ΔTL, with greater attrition shown by Lean and Hard birds.

### Current experiment

The current experiment began when the birds were over 2 years old (range 978–1044 days), by which time two birds of the original cohort (including one of the birds with missing ΔTL) had died. Two replicates of eight birds and two replicates of seven birds were sequentially caught from the aviary and moved to individual cages in our experimental room, keeping natal families together and thus balancing testing order by developmental treatment. One bird did not consistently peck keys and did not complete the impulsivity task.

Birds were housed in individual cages that served as both operant chambers and home cages for the entire experimental period. Cages were fitted with a panel consisting of three illuminable pecking keys and a feeder trough connected to a pellet dispenser delivering 45 mg grain-based rodent pellets (TestDiet, Richmond IN, USA) as described elsewhere (Feenders and Bateson 2013). Cages measured 100 × 45 × 45 cm, with two perches and plastic baths and the same ambient conditions as the aviary. While in individual cages, birds were food deprived from 17:00 until testing began the next morning at 07:00. Water was always available ad libitum. Operant sessions ended at 11:00 each day where baths and ad libitum food of the same type as in the aviaries were made available until 17:00. Birds, therefore, had 14 h of food deprivation prior to each operant session.

Operant panels were controlled by a remote computer using the Whisker experimental control system (Cardinal and Aitken 2010) and behavioural tasks were programmed in Microsoft Visual Basic 6.0.

### Training

Operant training procedures followed those outlined in Feenders and Bateson (2013). First, birds were auto-shaped to peck the centre amber key for a food reward. Birds then progressed to a variable number of days of operant training which ended once they successfully pecked the centre amber key for a food reward on at least 50% of the 80 trials presented in any one daily experimental session. On successful completion of operant training, birds moved on to the impulsivity task. At this point, we weighed the birds to calculate body condition, the residual of body mass after controlling for tarsus length.

### Impulsivity task

Birds made repeated simultaneous choices between a smaller sooner and a larger later food reward. Pecking a key illuminated in red produced one 45 mg pellet after a 3-s delay (the short-delay option). Pecking a key illuminated in green produced two 45 mg pellets after an 8-s delay (the long-delay option). We did not counterbalance the assignment of colours to options; as this was an individual-difference study, it is critical that all birds are making the same choice. The delays were chosen based on previous studies (Bateson et al. 2015; Nettle et al. 2015a) so as to be within the interval of indifference points of starlings, so that some birds would have an overall preference for the short-delay option, and some for the long-delay option.

Daily sessions consisted of 120 trials, divided into 30 blocks of four. Sessions ended either when 120 trials were completed or when 4 h had elapsed, whichever was sooner (birds finished a mean ± SE of 21 ± 0.22 blocks/day). Each block consisted of two forced trials (i.e. trials on which only one of the options was available) to ensure all birds experienced both options, followed by two choice trials (i.e. trials with both options available simultaneously). On forced trials, following a response to the amber key, the amber light extinguished and either a red or green light appeared on the right or left key. A single peck to this light initiated the start of the programmed delay. Following the programmed delay, a single further peck was required to extinguish the key light and initiate the delivery of reward (one pellet per second). During reward delivery, the hopper light was illuminated. Following the final pellet delivery, the inter-trial interval (ITI) began. Within each block, the two forced trials were chosen pseudo-randomly such that there was always one of each type (short and long delay), with one being presented on each side. Choice trials were identical to forced trials with the exception that following the initiation peck, both side keys were illuminated (one in red and one in green). A single peck indicated the bird’s choice and resulted in the non-chosen light being extinguished. In choice trials, the side on which each colour appeared was randomly chosen on each trial. The total duration of the ITI plus the chosen delay was always 120 s, to make the frequency of reward independent of the option chosen. Birds ran 7 days a week and all completed 10 days of the impulsivity task.

### Estimation of stability and final data inclusion

We calculated the proportion of short-delay choices on the choice trials of the impulsivity task by day and bird. Birds had higher proportions of short-delay choices on day 1 (mean 0.72, SD 0.15) than day 10 (mean 0.59, SD 0.24; paired *t* test: *t*_28_ = 2.54, *p* = 0.02). To estimate the stable level of preference, we, therefore, excluded the first 4 days (this number was determined by inspection of daily proportions of short-delay choices). The difference between birds’ proportions of short-delay choices on day 5 (i.e. the first day of the data now included; mean 0.67, SD 18) and on day 10 (mean 0.59, SD 0.24) was not significant (paired *t* test: *t*_28_ = 1.64, *p* = 0.11). We calculated the correlation coefficients between day and proportion of short-delay choices for each bird when including the data from days 5 to 10 only. After Bonferroni correction for carrying out 29 tests, 28 correlations were non-significant and 1 was significantly negative. All subsequent analyses are thus based on using the data for each bird from day 5 to day 10 inclusive. This results in a mean of 247.9 included choices per bird (minimum 193, maximum 215, SD 32.36).

### Statistical analyses

We used the proportion of completed choice trials on which the bird chose the short delay as the measure of impulsivity. We validated this measure by correlating the proportion of short-delay choices in the choice to the mean log-latency to peck the short-delay key on the fixed trials. Birds that assigned a greater value to the short-delay option should have lower latencies to peck the short-delay key when it was the only one illuminated (Bateson and Kacelnik 1995). There was indeed a strong negative partial correlation between the proportion of short-delay choices in the choice trials and the mean log-latency to peck the short-delay key in forced trials (partial correlation *r*_26_ = − 0.86, *p* < 0.001 after controlling for mean log-latency to peck the long-delay key in forced trials). An alternative to computing each bird’s proportion of short-delay trials is to take trial as the unit of analysis and model the outcome (short or long) using generalized linear mixed models with binomial error structure and a logit link function. This more complex analysis produces the same conclusions as the analysis by bird that we report below.

For the main analyses, linear mixed models were fitted using R package ‘afex’ (Singmann et al. 2018), with a random effect of natal nest to take account of the sibling relationships amongst birds. Fixed predictors were as specified in Table 1. We used separate models for the analysis of whether ΔTL predicted impulsivity, and whether the developmental treatments predicted impulsivity. This is because, since the developmental treatments affected ΔTL in these birds, ΔTL is potentially on the causal pathway, making the effects of developmental treatments in a model also including ΔTL hard to interpret.

**Table 1:** Summary of main models predicting proportion of short-delay choices

Model	*N*	Fixed predictors	*B*	se (*B*)	LRT	*p* value
1	25	ΔTL	0.103	0.369	0.08	0.78
		Body condition	− 0.039	0.283	0.02	0.90
		ΔTL × body condition	0.065	0.300	0.04	0.85
		Sex (male)	0.482	0.426	1.06	0.30
2	29	Amount	− 0.221	0.142	2.29	0.13
		Effort	0.233	0.147	2.41	0.12
		Body condition	− 0.305	0.181	2.13	0.14
		Amount × body condition	− 0.073	0.142	0.26	0.61
		Effort × body condition	− 0.487	0.140	8.40	0.004
		Amount × effort	0.405	0.130	9.81	0.002
		Amount × effort × body cond.	− 0.060	0.144	0.17	0.68
		Sex (male)	0.693	0.338	3.64	0.06

Continuous variables were standardized prior to model fitting

All continuous variables were standardized prior to analysis to facilitate comparison to the findings of earlier studies. Estimation was by maximum likelihood except for the testing of random effects, for which the model was estimated by reduced maximum likelihood. Significance testing was by likelihood ratio test (LRT), using the package ‘RLRSim’ (Schiepl et al. 2008) for the testing of the random effect. An *α* level of 0.05 was used throughout.

Birds that made a higher proportion of short-delay choices also completed more trials (*r*_27_ = 0.61, *p* < 0.01). This may have been because more impulsive birds, by choosing the short-delay option more often, received less food over the course of sessions, and consequently were more motivated to work. Some support for this contention comes from the fact that there was a marginally non-significant negative correlation between proportion of short-delay choices and mean log-latency to initiate trials on illumination of the amber light (*r*_27_ = − 0.37, *p* = 0.05). We did not control for total number of trials completed in the models, as we interpret the number of trials completed as partly a consequence of the bird’s impulsivity, and hence inappropriate to control for where impulsivity is the outcome variable. However, including total number of trials as an additional predictor in the models reported below does not alter any conclusion.

For the meta-analysis objective, we extracted parameter estimates and standard errors from analogous models using the data from Bateson et al. (2015) and Nettle et al. (2015a) as well as the present experiment. These were entered into fixed-effects meta-analyses using the ‘metafor’ package (Viechtbauer 2010).

### Data and code availability

All analyses were conducted in R (R Core Team 2016). The raw data files and R script are available at the Zenodo repository (10.5281/zenodo.1441061).

## Results

### Distribution of impulsivity and association with total trials completed

Birds varied from 0.24 to 0.98 (mean 0.63, SD 0.18) in their proportion of short-delay choices. In a model with proportion of short-delay choices as the outcome and no predictors other than an intercept and the random effect of natal nest, natal nest explained 17% of the variation in impulsivity. However, this was not significantly greater than chance according to the LRT (LRT = 0.74, *p* = 0.17).

We correlated the proportion of short-delay choices in this experiment to the proportion of risky choices made by the same birds in the study of risk preference by Andrews et al. (2018). The correlation between the two measures was not significantly different from zero (*r*_27_ = − 0.06, *p* = 0.76).

### ΔTL and body condition as predictors of impulsivity

In a model with ΔTL, body condition and their interaction as fixed predictors, neither ΔTL, body condition, nor their interaction significantly predicted proportion of short-delay choices (Table 1, model 1).

**Fig. 1:**
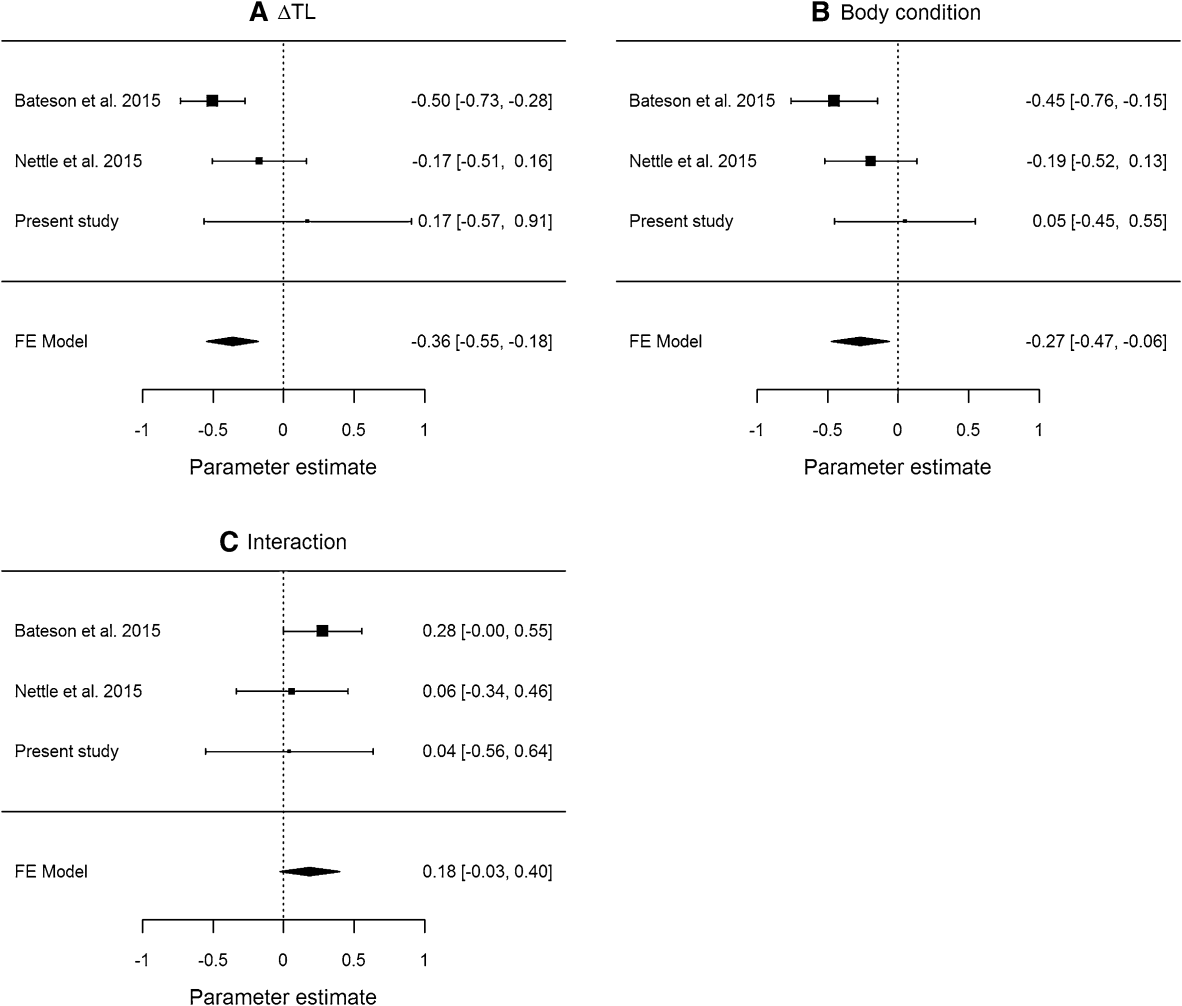
Forest plots from meta-analyses of three starling impulsivity experiments combined, showing the parameter estimates and 95% confidence intervals for each study and overall. **a** The association between ΔTL and impulsivity. **b** The association between body condition and impulsivity. **c** The interaction between ΔTL and body condition in predicting impulsivity. All parameter estimates are standardized

To compare these results with the earlier findings, we fitted the models exactly equivalent to model 1 in Table 1 to the data from the two earlier studies (Bateson et al. 2015; Nettle et al. 2015a) and performed meta-analysis separately for the association of impulsivity with ΔTL, the association of impulsivity with body condition, and the interaction between ΔTL and body condition (Fig. 1). As Fig. 1 shows, despite completely null findings from the present experiment, when the data from the three studies were combined, there was still overall support for a negative association of ΔTL with impulsivity (that is, more telomere loss associated with greater impulsivity), and for a negative association of body condition with impulsivity (that is, lighter body condition associated with greater impulsivity). The confidence interval for the combined estimate of the interaction term crossed zero.

### Developmental treatments as predictors of impulsivity

**Fig. 2:**
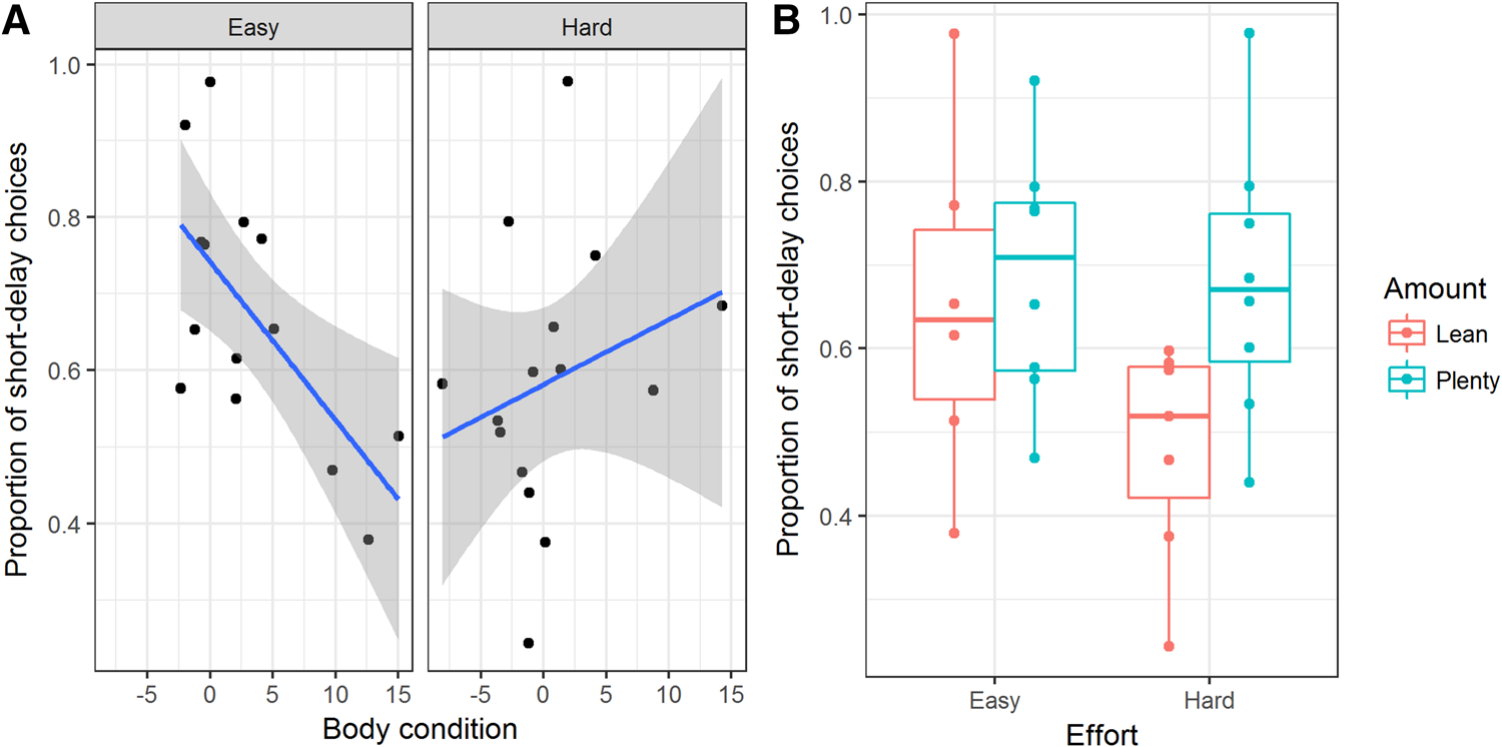
Summary of findings on developmental treatments and body condition as predictors of impulsivity. **a** Proportion of short-delay choices in relation to body condition and early-life begging Effort. Shaded areas represent 95% confidence intervals. **b** Boxplot of the proportion of short-delay choices in relation to early-life begging Effort and early-life food Amount

In a model with the two developmental treatments, Amount and Effort, in addition to body condition, as fixed predictors (Table 1, model 2), there were significant interactions between Effort and body condition, and also between Effort and Amount. The first of these interactions was created by the predicted association between body condition and impulsivity (lighter birds being more impulsive) being present in the birds that had experienced the Easy treatment (*r*_12_ = − 0.67, *p* = 0.009), but absent in birds that had experienced the Hard treatment (*r*_12_ = 0.26, *p* = 0.36; Fig. 2a). The second interaction was due to birds that had experienced both Hard begging effort and Lean amount having lower proportions of short-delay choices than other groups (Fig. 2b; estimated marginal means (standard errors): Lean-Hard: 0.50 (0.07); Lean-Easy: 0.78 (0.08); Plenty-Hard: 0.68 (0.05); Plenty-Easy 0.67 (0.05)).

## Discussion

We examined the associations between developmental experience, developmental telomere attrition, energetic reserves, and impulsivity in adulthood in a cohort of hand-reared starlings. Our impulsivity task differed from earlier studies in that it did not involve an adjusting delay, and hence excluded the possibility of its results being influenced by risk preference. Our fixed-alternative task produced a striking amount of variation in impulsivity, with birds varying from a near-total preference for the short delay, through to a fairly strong preference for the long delay. We found a modest but non-zero familial variance component (17%). Though this was very similar to the magnitude of the familial component in the earlier study by Bateson et al. (2015; 14%), it was not sufficient with this sample size to formally reject the null hypothesis that there might be no familial effects on impulsivity.

We did not replicate the findings from Bateson et al. (2015) that greater developmental telomere attrition predicts greater impulsivity in adulthood, or that lower energetic reserves overall, as measured by lower body condition, predicts greater impulsivity. However, we found evidence for developmental experience being related to adult impulsivity. Specifically, the predicted association between body condition and impulsivity was present in the birds that had experienced low begging effort, but abolished amongst those who had experienced high begging effort. Moreover, birds that had experienced high begging effort in combination with low food amount were significantly less impulsive than other birds.

Our failure to replicate the pattern observed in the study by Bateson et al. (2015) in respect of developmental telomere attrition is difficult to interpret. On the one hand, it is quite possible the discrepancy represents normal sampling variability: either the effects of the original study were atypically strong, or ours are atypically weak, or both. Only many more studies would be able to adjudicate in this regard. Combining the three studies done to date meta-analytically still lends some support to the conclusion that birds that undergo greater developmental telomere attrition are more impulsive as adults, and that birds with lower energetic reserves are more impulsive overall. This is because the associations reported by Bateson et al. (2015) were quite strong and had relatively small standard errors, and the associations in the study by Nettle et al. (2015a), although non-significant, were in the same direction. The present null results thus do not completely offset the earlier patterns when all three studies are combined.

However, our impulsivity measure was also different from the earlier two studies, in that it avoided any possibility of contamination by individual differences in risk preference. We confirmed the independence of impulsivity as we measured it here from risk preference by finding an almost zero correlation between proportion of short-delay choices in this experiment, and proportion of risky choices in an experiment on risk preference the same birds (Andrews et al. 2018). In view of the different methodologies, it is not clear that the present results should be simply combined with those of the two earlier studies. Without wishing to interpret difference of significance as significance of difference, it is interesting that the results of the present experiment are so unlike those of Bateson et al. (2015). If it were the case that the indifference point in the adjusting-delay impulsivity procedure is also affected by risk aversion, and birds that undergo greater developmental telomere attrition are more risk averse (as in Andrews et al. 2018), then this could produce an association between impulsivity based on the adjusting-delay indifference point and developmental telomere attrition. However, until developmental telomere attrition, risk preference, and impulsivity assessed both ways are measured on the same individuals, this remains a conjecture. Unfortunately, this was not possible in the present birds due to the length of time involved in running any one of these operant paradigms.

The observed effects of early-life treatments on impulsivity were not predicted a priori. Indeed, one of the main conclusions arising from our work in earlier cohorts of birds (particularly Nettle et al. 2015a) was that developmental telomere attrition has greater predictive value for adult behavioural phenotype than the developmental treatments to which the birds were exposed. Thus, our prior expectation was to find developmental telomere attrition effects and not developmental treatment effects, the opposite of what we observed. However, the interaction we found between early-life begging effort and body condition does corroborate other evidence we have from these same birds that the regulation of energetic reserves in adulthood is affected by Hard begging effort (Dunn et al. 2018). Dunn et al. (2018) showed that the Hard birds maintain lower masses for their skeletal size, and defend their rate of food intake more strongly, than the Easy birds. In the present experiment, the Hard birds, particularly those who also experienced low food Amount, chose the long-delay option more often. This resulted in a higher rate of energy intake through the sessions (because the total length of the delay plus inter-trial interval was fixed, always choosing the long-delay option results in twice the rate of energy intake over the session). In general, starlings fail to maximize their rate of intake over the course of the session, because they fail to include the inter-trial interval in their computation of the value of options (Bateson and Kacelnik 1996). However, it may be that the Hard birds were more influenced by the rate of intake than is typical. Amongst the Easy birds, as energy reserves dropped, they switched more to the short-delay option, as previous findings predicted. This was not, however, the case for the Hard birds, whose energy reserves were also lower overall. Thus, we have indirectly confirmed in this experiment the contention of Dunn et al. (2018) that high early-life begging effort produces an alternate foraging strategy based on allowing fat reserves to be low but defending the rate of energy intake (see Dunn et al. 2018 for further discussion of why begging effort may have this effect). However, although the treatment findings are interpretable post hoc, note that they are not in the direction of the most adverse developmental conditions leading to the greatest impulsivity in adulthood. This was the direction of the effects in a previous study in rats finding evidence that developmental stress influences adult impulsivity (Gondré-Lewis et al. 2016). Here, the Lean-Hard group experienced the most adverse conditions, and was the least impulsive.

In conclusion, using a modified impulsivity task for starlings in which neither option is variable in the bird’s experience, we did not find evidence for an association between developmental telomere attrition and impulsivity. We did find that energetic reserves were associated with greater impulsivity, but only in those birds who experienced a relatively low level of early-life begging effort. Birds that had experienced the combination of high begging effort and restricted food supply in early life were less impulsive overall.
